# A Comparison of the Pulmonary Valve, Main Pulmonary Artery, and Branch Pulmonary Artery Measurements by Echocardiography and Computed Tomography Scan

**DOI:** 10.7759/cureus.16075

**Published:** 2021-06-30

**Authors:** Priyanka A Parikh, Binoy V Shah, Bhadra Trivedi, Viral B Patel, Shreya Desai, Somashekhar M Nimbalkar, Ajay G Phatak

**Affiliations:** 1 Department of Pediatrics, Pramukhswami Medical College, Karamsad, IND; 2 Pediatric Cardiology, Shree Krishna Hospital, Karamsad, IND; 3 Radiodiagnosis, Pramukhswami Medical College, Karamsad, IND; 4 Radiodiagnosis, Shree Krishna Hospital, Karamsad, IND; 5 Radiodiagnosis, Bhaikaka University, Karamsad, IND; 6 Central Research Services, Bhaikaka University, Karamsad, IND

**Keywords:** trans-thoracic echocardiography, pediatrics & neonatology, pediatric congenital heart disease, multidetector computed tomography, echocardiography - heart failure - valvular heart disease, intervention pediatric cardiology, pediatric clinical cardiology, cardiothoracic & vascular surgery research, computed tomography

## Abstract

Introduction: Congenital heart diseases (CHD) are one of the most commonly occurring congenital anomalies. Echocardiography is usually the initial investigation for suspected CHD. However, it is operator-dependent and limited by available chest windows. Multidetector computed tomography (MDCT) scan provides superior temporal and spatial resolution producing excellent cross-sectional anatomical images. MDCT is specifically helpful for pulmonary artery anomalies if not clearly visible on an echocardiogram.

Objective and methods: The study aims to compare measurements of branch pulmonary arteries, pulmonary valve, and main pulmonary artery obtained from trans-thoracic echocardiography measurements and MDCT. Forty-nine patients younger than 17 years of age underwent MDCT, and an echocardiogram was included in the study. The measurements of the pulmonary valve, main pulmonary artery, and branch pulmonary arteries were measured on MDCT and echocardiogram.

Results: Bland-Altman analysis revealed the mean difference (95% confidence limits) in measurements of diameter between echocardiogram and MDCT for the right pulmonary artery, left pulmonary artery, pulmonary valve, and main pulmonary artery, which were -0.5 (-3.1, 2.2) mm, -0.6 (-3.3, 2.1) mm, 0.7 (-2.5, 3.9) mm, and 1.2 (-6.9, 4.5) mm, respectively.

Conclusion: The analysis revealed acceptable agreement in measurements of the pulmonary valve, main pulmonary artery, and branch pulmonary arteries obtained from MDCT and echocardiogram. The difference was marginally more for the main pulmonary artery compared to the pulmonary valve and branch pulmonary arteries.

## Introduction

Congenital heart diseases (CHD) comprise 8/1000 live births and account for a high prevalence of infant mortality worldwide [1,2]. Trans-thoracic echocardiography (TTE) is the most preferred investigation for evaluating CHD; however, it is operator-dependent and limited by the acoustic window. Over recent years, the TTE machines have undergone a significant upgrade in terms of technological advances. Hence, the image quality of 2D TTE is reliable and reproducible in experienced hands [3]. It has the advantage of being non-invasive and radiation-free. The TTE has Doppler capabilities and can track blood cells passing through the vessels. Thus studying the anatomic and hemodynamic details without intravenous contrast is possible.

Multidetector computed tomography (MDCT) scan has been increasingly utilized for imaging the heart for various congenital and acquired cardiac anomalies. It gives superior temporal and spatial resolution producing excellent cross-sectional anatomical images. Thus, it is considered the gold standard to study static anatomic details for the cardiac structures in the pediatric population [4]. With advancements in the MDCT scan technology, axial data can generate non-axial images, such as sagittal and coronal, using multiplanar reformation (MPR). Software analysis tools like maximum intensity projection (MIP) and volume rendering (VR) techniques have improved diagnostic capabilities. Precise three-dimensional imaging obtained from the MDCT scan help in planning the treatment for congenital and acquired cardiac pathologies [5]. The drawbacks of the MDCT scan are the inevitable radiation doses and the use of a contrast medium, which is essential to assess the anatomy of smaller vessels.

Accurate preoperative assessment of pulmonary artery anatomy influences the surgical approach and aids in treatment planning [6]. The anatomy of the pulmonary valve, main pulmonary artery, and branch pulmonary arteries are the significant components of evaluation in cases of suspected congenital heart disease as per recommended guidelines [7]. Adequate information is received from the echocardiogram most of the time. Computed tomography (CT) scan pulmonary angiogram enhances the accuracy of measurements and compliments the echocardiogram findings. The study aims to assess whether TTE measurements and MDCT measurements of branch pulmonary arteries, pulmonary valve, and main pulmonary artery are comparable.

## Materials and methods

This retrospective study was approved by the Institutional Review Board based on a medical record review. Forty-nine pediatric patients who underwent MDCT pulmonary angiogram and echocardiogram were included in the study. Each case was discussed among the authors to ensure that the patients’ clinical condition had not changed between the MDCT and TTE, which would otherwise affect the sizes of great vessels. If either of the study modality MDCT or TTE were sub-optimal or missing, the cases were excluded. Information about the demographic profile, including the cardiac disease, was gathered without personal identifiers. The TTE evaluation was performed using the Siemens Acuson x300 USG machine (Siemens, Munich, Germany) using Siemens Acuson P8-4 phased array sector. It was performed by the same pediatric cardiologist every time following a standard protocol in a dedicated echocardiogram lab. Whenever a child was not cooperative and sedation was required, Trichlophos oral solution was used with 50 mg/kg single dose. Patients were observed in the nursing station in the outpatient department (OPD) area until the child was fully awake. The measurements of the pulmonary valve were done using the left parasternal long-axis view. The main pulmonary artery and branch pulmonary arteries were measured in the left parasternal short-axis view in systole as per recommendation [8]. The maximum diameter was considered for measurements. The MDCT scans were performed on GE Healthcare Optima CT 660 (GE Healthcare, Chicago, Illinois). The normal value of serum creatinine was mandatory before each MDCT scan.

**Figure 1:**
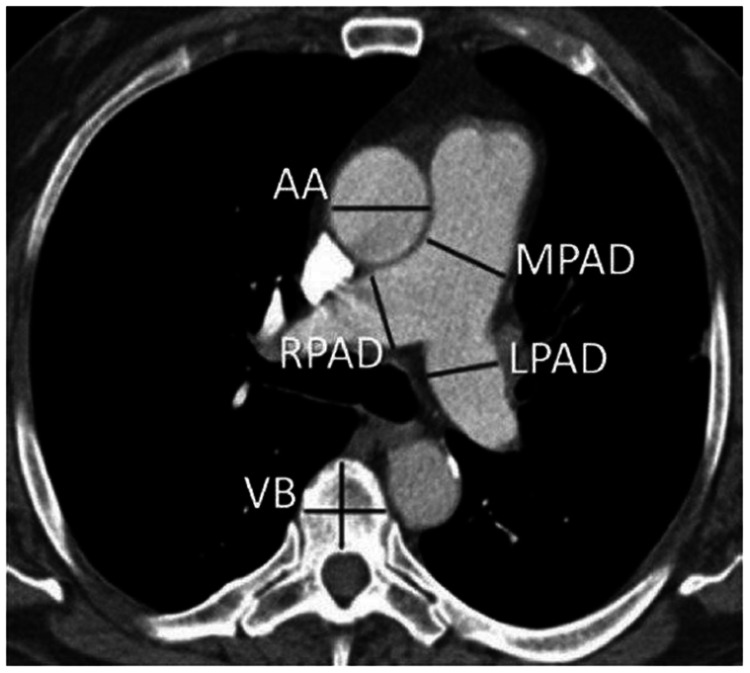
Axial image at the level of bifurcation of a main pulmonary artery AA, Ascending aorta; MPAD, main pulmonary artery diameter; LPAD, left pulmonary artery diameter; RPAD, right pulmonary artery diameter; VB, vertebral body.

A combination of injectable ketamine, midazolam, and glycopyrrolate was used for sedation during the MDCT scan as per the protocols followed by the pediatric cardiac intensivist. Patients were examined in a supine position, and all scans extended from the nape to the aortic bifurcation in a craniocaudal direction to assess abdominal vascular structures and viscera. Non-ionic iodinated contrast material (Vizipaque 270 mg/ml) was given intravenously by a pressure injector at a rate usually varying from 2 ml/s to 2.5 ml/s. The total dose was kept at 2 ml/kg of body weight. Saline bolus injection followed contrast with the same volume and rate. Since the access route was not fixed in all patients, e.g., arm or leg, a modified bolus tracking setting was used depending on access. The scan was initiated as soon as the contrast was visible in the upper part of descending aorta. The acquisition parameters used for CT examinations were as follows: 80 kVp, 100-240 mAs, and 0.6 mm slice thickness. The gantry rotation time was 0.5 seconds, the collimation was 128 × 0.6 mm, and the matrix was 512 × 512 mm. Region of interest (ROI) was set in the ascending aorta keeping Hounsfield units (HU) of 80. The 2D and 3D reconstructions in different planes were performed in maximum intensity projections. The measurements of the pulmonary valve, the main pulmonary artery, and branch pulmonary arteries were interpreted by a single radiologist with 14 years of experience after a review of each scan using a dedicated advanced workstation. The measurements of the pulmonary valve, the main pulmonary artery, and branch pulmonary arteries used in this study were derived after a review of each scan using the dedicated advanced workstation as per standard practice [9]. The radiologist was blinded with findings and measurements of TTE. On axial images at the level of bifurcation of a main pulmonary artery, the transverse diameter of the main pulmonary artery was taken in the image where the whole pulmonary trunk from annulus till bifurcation was visualized utilizing angulated multiplanar reformation (Figure 1) [10]. The branch pulmonary arteries were measured on axial images at a level of maximum diameter before branching (Figure 1). The pulmonary valvular level measurements were done from acquired CT image data by postprocessing and obtaining sagittal MIP reformation at a level where the right ventricle and main pulmonary artery were visualized [11].

Statistical analysis

Descriptive statistics [mean (SD), frequency (%)] were used to depict the profile of participants. The agreement between different parameters was assessed using Bland-Altman analysis. The normality of the differences was assessed by histogram. The agreement was presented as a mean difference (95% confidence limits). The analysis was performed using Stata 14.2 (StataCorp, College Station, TX, USA).

## Results

A total of 49 patients (34 M, 15F) were enrolled in the study. Of these, 69% were males, and the mean [Median (Q1, Q3)] age of participants was 46.5 [18 (5,81.3)] months. Almost half [22 (46.9%)] of the participants were less than one-year old, while another quarter [13 (26.5%)] of them were between one and five years of age. Cyanotic heart disease was present in 35 children who underwent MDCT scans in this study population. Tetralogy of Fallot, in its spectrum for atresia to absent pulmonary valve, was found in 48% of the patients (Table 1).

**Table 1:** Cardiac and pulmonary lesions in patients who underwent a CT scan and echocardiogram CT, Computed tomography.

Diagnosis of congenital heart disease	N	%
Tetralogy of Fallot with pulmonary stenosis	17	34.69
Tetralogy of Fallot with pulmonary atresia	4	8.16
Tetralogy of Fallot with absent pulmonary valve	3	6.12
Total anomalous pulmonary venous connection	3	6.12
Scimitar syndrome	1	2.04
Atrial septal defect	3	6.12
Single ventricle with pulmonary stenosis	4	8.16
Pulmonary arteriovenous malformation	1	2.04
Pulmonary stenosis with intact ventricular septum	1	2.04
Single ventricle pulmonary hypertension	2	4.08
Ventricular septal defect with coarctation	1	2.04
Coarctation with patent ductus arteriosus	2	4.08
Double outlet right ventricle with pulmonary stenosis	2	4.08
Branch pulmonary artery stenosis	1	2.04
Single ventricle pulmonary atresia	1	2.04
Ventricular septal defect	1	2.04
Pulmonary embolism	1	2.04
Kawasaki syndrome	1	2.04
Total	49	

**Figure 2:**
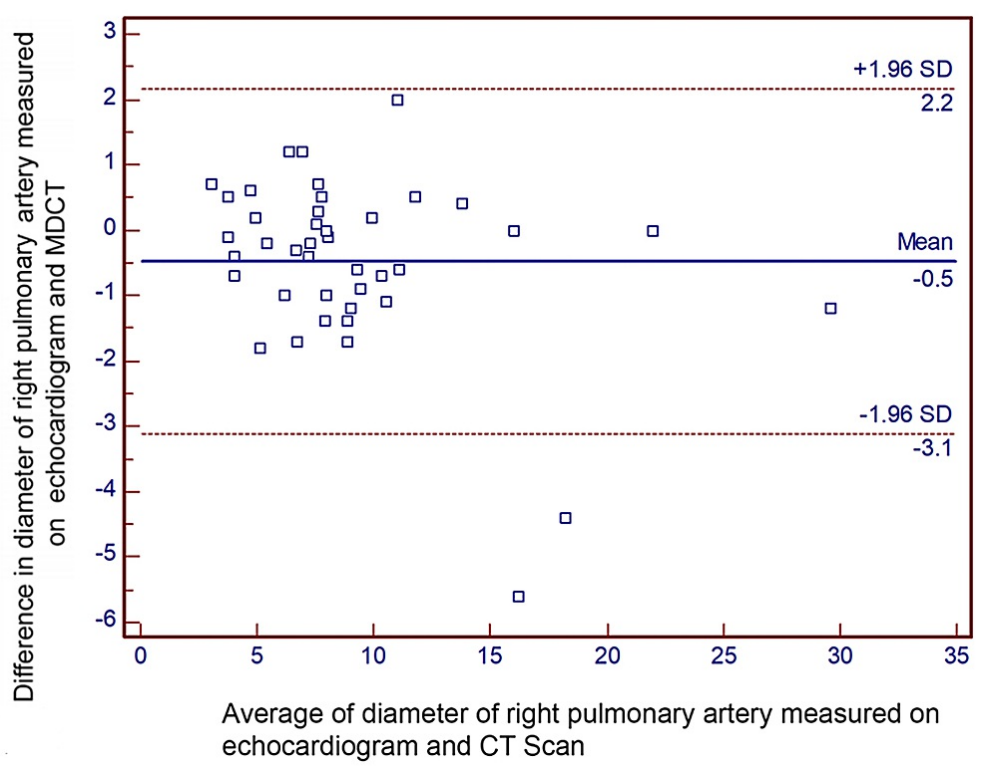
Bland-Altman analysis for the diameter of the right pulmonary artery

**Figure 3:**
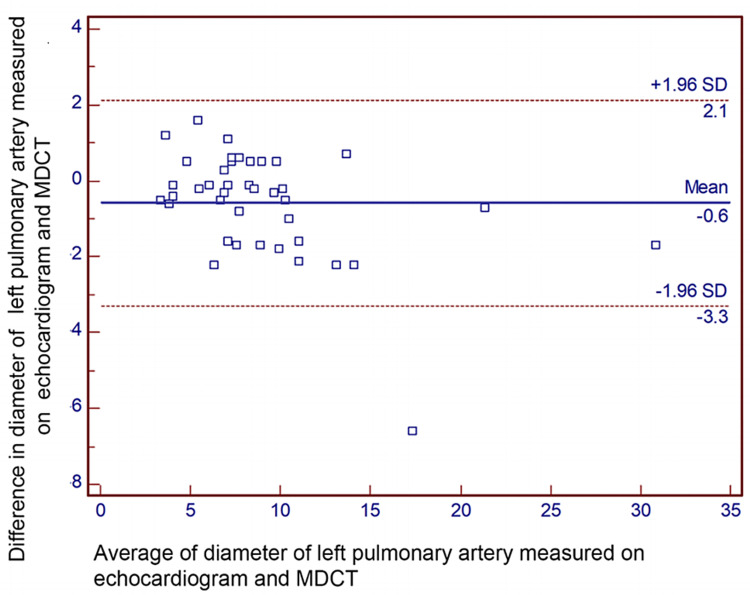
Bland-Altman analysis for the diameter of the left pulmonary artery

**Figure 4:**
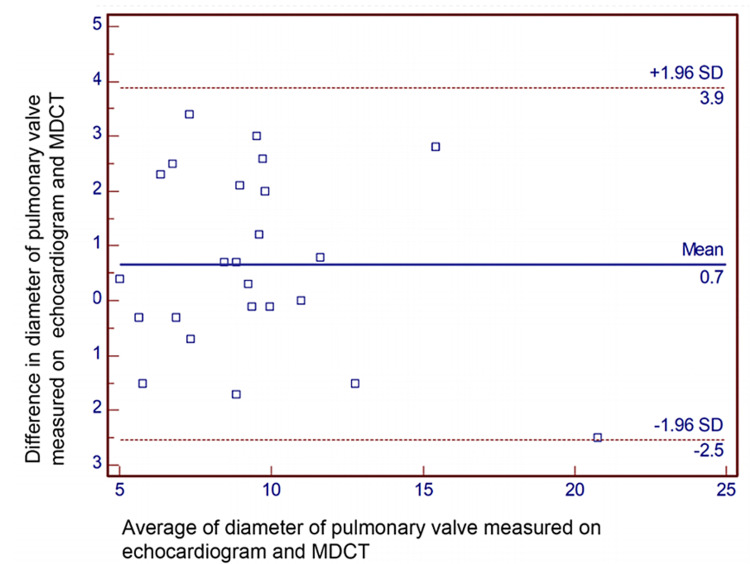
Bland-Altman analysis for the diameter of the pulmonary valve

**Figure 5:**
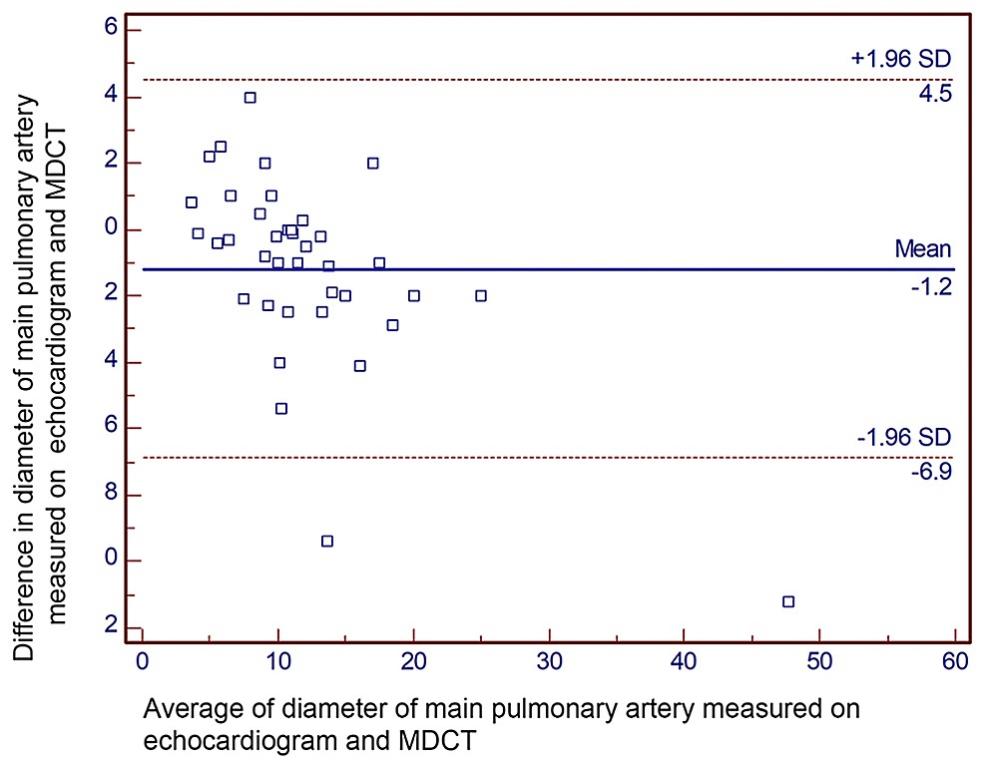
Bland-Altman analysis for the diameter of the main pulmonary artery

Bland-Altman analyses revealed the mean difference (95% confidence limits) of diameter for the right pulmonary artery, measured on TTE and MDCT, was -0.5 (-3.1, 2.2) mm (Figure 2). Similarly, the mean difference (95% confidence limits) for the diameter of the left pulmonary artery was -0.6 (-3.3, 2.1) mm (Figure 3). The mean difference (95% confidence limits) diameter of the pulmonary annulus as measured on TTE and MDCT was 0.7 (-2.5, 3.9) mm for the diameter of the pulmonary valve (Figure 4). The difference was highest for the main pulmonary artery, 1.2 (-6.9, 4.5) mm (Figure 5). The differences were without any upward/downward trend.

It was observed that out of 49 patients, 13 patients did not require surgical intervention. Out of 36 patients who were advised surgical intervention, 29 patients underwent surgeries. In 27 patients, the surgical plan remained the same after the MDCT scan as it was decided on an echocardiogram prior to the CT scan. In two patients, a surgical plan could not be decided on an echocardiogram alone; their plan was finalized after the CT scan.

## Discussion

Most children born with congenital heart disease require intervention, and the nature of the intervention is based on the anatomic details and hemodynamic conditions. TTE is used as a diagnostic tool for congenital heart disease since 1967 [12,13]. It is well established that TTE provides sufficient information for intracardiac structures, i.e., cardiac valves, chamber dimensions, intracardiac shunts, and hemodynamics [14,15]; however, due to its limitation with acoustic window, the extracardiac structures, i.e., distal pulmonary arteries, branch of the aorta, and individual pulmonary veins, are not very well visualized. Thus MDCT scan has been a preferred modality for pulmonary artery details when TTE is unequivocal [16].

It was noted in our study that for those who required surgical intervention - corrective or palliative - the provisional surgical plan based on echocardiogram did not change after the MDCT scan if the echocardiogram study provided sufficient information. There were two cases (a five-month-old male child with Scimitar syndrome and a 12-day-old male child with tetralogy of Fallot [TOF] with pulmonary atresia) where the TTE findings were unequivocal for surgical planning and MDCT provided details for surgical planning.

The study showed that the diameter of the pulmonary valve, main pulmonary artery, and branch pulmonary arteries measured on TTE and MDCT pulmonary angiogram were comparable.

The pulmonary valve annulus is one of the determinants to decide the type of corrective surgery for TOF, i.e., intracardiac repair with valve-sparing or trans-annular patch. The annular sparing surgeries carry preferable early surgical outcomes [6]. Our study shows the difference between the mean size of pulmonary valve annulus measured between echocardiogram and CT scan to be 0.7 mm, similar to the study by Sathio et al. [17].

Complete correction for TOF cannot be done if the branch pulmonary arteries are smaller in size. Z score of pulmonary arteries is essential to decide the adequacy of branch pulmonary arteries for complete repair [18]. Earlier, cardiac catheterization and angiography were considered the gold standard for measuring right ventricular outflow tract components - starting from the subpulmonary region, pulmonary valve, main pulmonary artery, and branch pulmonary arteries [19]. Multidetector row computed tomography gained popularity as it is a significantly less invasive procedure while giving measurements comparable to an angiogram. A study of 56 MDCT scans for measurement of pulmonary arteries showed an excellent correlation between MDCT and conventional angiogram [20]. The current study results proved the comparability of measurements of the pulmonary arteries between TTE and MDCT. For measurement of pulmonary arteries using MDCT and TTE, the mean difference was being less than 1 mm for both branch pulmonary arteries, thus suggesting TTE be the first-line investigation and MDCT to be reserved for some instances due to its associated risk of radiation.

A 13-year review of surgical outcomes of 598 children of TOF suggested that TTE alone was sufficient to pick up anomalous coronary anatomy without the need for additional imaging [21]. The proportion of TOF patients undergoing cardiac surgery based on only TTE was 11%, which went up to 82% between 1990 and 1995 due to advancements in technology [21]. MDCT scan remains the gold standard to study the complex coronary anatomy when an echocardiogram is not very informative. MDCT is also the modality of choice for complex anatomies such as aortopulmonary collaterals in the pulmonary atresia setting where native branch pulmonary arteries are either diminutive or may be completely absent. The treatment plan for such children needs MDCT [22].

The current study also showed good parity between the mean measurements of the pulmonary valve, measured on TTE and MDCT with a mean difference of 0.7 mm. Most of our TTE was done under sedation allowing the pediatric cardiologist to perform a detailed echocardiogram.

The pulmonary valve is situated behind the sternum, sometimes making it difficult to assess on TTE. Due to its anterior position, trans-esophageal echocardiogram (TEE) also does not yield satisfactory images. In such cases, MDCT or MRI is necessary for evaluating the pulmonary valve [23]. TTE is the primary modality of choice to assess hemodynamics, and thus, it is used to assess the severity of pulmonary stenosis or regurgitation, while MDCT is necessary to evaluate the geometry and morphometry of RVOT [24].

TTE machines have undergone significant upgrades, starting from M Mode to two-dimensional imaging and three-dimensional imaging [25]. Newer echocardiogram machines are even equipped with true confocal image (TCI) and adaptive contrast enhancement (ACE) technologies. They help in acquiring high-resolution images and also help in differentiating between different tissues [26].

Limitation

The sample size and the retrospective nature of the study itself might have some inherent biases. The echocardiogram and MDCT both are operator-dependent. Similar results may not be reproducible at another center. CT scan also excels in studying the lobar branches and beyond the pulmonary tree.

## Conclusions

We conclude that the measurements of the pulmonary valve, main pulmonary artery, and branch pulmonary arteries in children obtained from the TTE study are comparable with MDCT. Hence, MDCT can be reserved for certain complex cases. It can help reduce exposure to radiation due to MDCT and reduce the treatment cost while ensuring the quality of care. We believe that more emphasis should be placed on performing a comprehensive echocardiogram by an experienced pediatric cardiologist.
